# Breast carcinoma with 21-gene recurrence score lower than 18: rate of locoregional recurrence in a large series with clinical follow-up

**DOI:** 10.1186/s12885-017-3985-y

**Published:** 2018-01-06

**Authors:** Gulisa Turashvili, Edi Brogi, Monica Morrow, Maura Dickler, Larry Norton, Clifford Hudis, Hannah Y. Wen

**Affiliations:** 1 Department of Pathology, Memorial Sloan Kettering Cancer Center, 1275 York Avenue, New York, NY 10065 USA; 2 Department of Surgery, Memorial Sloan Kettering Cancer Center, New York, NY 10065 USA; 3 Department of Medicine, Memorial Sloan Kettering Cancer Center, New York, NY 10065 USA

**Keywords:** Breast cancer, Estrogen receptor positive, Early stage, 21-gene recurrence score assay, Low risk, Locoregional recurrence

## Abstract

**Background:**

The 21-gene recurrence score (RS) assay determines the benefit of adding chemotherapy to endocrine therapy for patients with early stage, estrogen receptor (ER)-positive, HER2-negative breast cancer. The RS risk groups predict the likelihood of distant recurrence and have recently been associated with an increased risk of locoregional recurrence (LRR). This study analyzed clinicopathologic features of patients with low RS and LRR.

**Methods:**

In our institutional database, we identified 1396 consecutive female patients with lymph node negative, ER+/HER2- invasive breast carcinoma and low RS (<18) results, treated at our center from 2008 to 2013. We collected data on clinicopathologic features, treatment and outcome.

**Results:**

The median patient age was 57 years (range 22–90). The median tumor size was 1.2 cm (range 0.3–5.8). Overall, 66.6% (930/1396) women were treated with breast conserving surgery (BCS) and radiation therapy, 3.4% (48/1396) with BCS alone, 29.7% (414/1396) with total mastectomy, and 0.3% (4/1396) with total mastectomy and radiation therapy. Most patients (84.8%; 1184/1396) received endocrine therapy alone, 12.1% (169/1396) were treated with chemotherapy plus endocrine therapy, and only 3.1% (43/1396) received no systemic therapy. At a median follow-up of 52 months, 0.9% (13/1396) of patients developed LRR. Sites of LRR included the ipsilateral breast (*n* = 8), chest wall (*n* = 3), axillary node (*n* = 1), and internal mammary node (*n* = 1). All patients with LRR had negative resection margins at the initial surgery. The rate of LRR in patients treated with adjuvant endocrine therapy alone was 0.7% (8/1184). All eight patients received standard local treatment. Three patients had lymphovascular invasion but no other significant risk factors for LRR were identified.

**Conclusions:**

Our study of node negative, ER+/HER2- breast cancer patients with low RS observed extremely low rates of LRR: 0.9% (13/1396) in the whole cohort and 0.7% (8/1184) in patients treated with endocrine therapy alone. As the largest series to date, we report detailed clinicopathologic data and clinical outcomes of this cohort and provide a comprehensive characterization of patients who developed LRR.

## Background

Multigene prognostic gene signatures developed in the last two decades have become an integral part of standard clinical management of breast cancer as they allow to identify patients at a higher risk of distant recurrence [1–5]. The 21-gene recurrence score (RS) assay (Oncotype Dx™, Genomic Health, Redwood City, CA) is the most used prognostic assay in the United States, recommended by National Comprehensive Cancer Network and the American Society of Clinical Oncology for patients with early stage, estrogen receptor (ER)-positive, HER2-negative breast cancer [6].

The RS assay utilizes reverse transcriptase polymerase chain reaction (RT-PCR) to quantify the expression of 16 cancer related genes normalized to the expression of five reference genes [3]. The resulting RS is a continuous variable on a scale of 0 to 100 which estimates the five year risk of distant recurrence in patients treated with tamoxifen and the benefit of adding chemotherapy to endocrine therapy [3, 4, 7]. Based on the RS values, breast cancer patients are stratified into three risk categories; low risk (RS < 18), intermediate risk (RS 18–30), and high risk (RS > 30) [3]. In patients with RS < 18, the benefit of chemotherapy is believed to be too small (2%) to outweigh the risks of secondary side effects. The clinical management of patients with a RS of 18 to 30 varies and includes endocrine therapy with or without chemotherapy depending on other clinicopathologic variables and patient’s choice. In contrast, patients with RS > 30 greatly benefit from chemotherapy due to a significantly increased risk (28%) of distant metastases reported by many studies [8–17]. The RS risk groups have recently been associated with an increased likelihood of locoregional recurrence (LRR) in several studies [18–20], including a large patient cohort from our institution [21], but the data remain limited.

As the largest series to date, we report detailed clinicopathologic data and clinical outcomes of consecutive female patients with lymph node negative, ER+/HER2- breast cancer and low RS (<18) treated at our institution and provide a comprehensive characterization of patients who developed LRR.

## Methods

### Study subjects

At our institution, all lymph node negative, ER+/HER2- invasive breast carcinomas measuring ≥0.5 cm are routinely evaluated with the 21-gene RS assay. In rare cases, testing of selected <0.5 cm tumors has also been requested by the clinician if patients are deemed medically suitable for chemotherapy and interested in receiving such treatment. We identified consecutive female patients with early stage, ER+/HER2- invasive breast carcinoma and low RS (<18) treated at our center between September 2008 and August 2013. Our study cohort consists of patients with negative lymph nodes (pN0(i+) and pN0) [22]. Male patients and tumors that failed testing for various technical reasons were excluded from the study.

We recorded clinicopathologic variables for all patients such as age at breast cancer diagnosis, tumor type and size, lymphovascular invasion (LVI), RS result, surgical and medical treatment, and clinical outcome. For multifocal ipsilateral invasive carcinomas, we recorded the size of the largest tumor and the highest RS result. For one patient with metachronous bilateral ER+/HER2- breast carcinomas with low RS, we only included data pertaining to the first tumor. We reviewed the institutional database and electronic medical records to identify patients with LRR and recorded sites of recurrence. The Institutional Review Board approved the study.

LRR was defined as the development of invasive breast cancer in the ipsilateral breast parenchyma, axilla, regional lymph nodes, chest wall or skin ≥6 months after the initial diagnosis [23]. The cut-off date for follow-up was September 1st, 2016. All data presented in this article are descriptive. No formal statistical analysis was performed due to the small number of LRR events.

## Results

### Patient cohort

We identified 1396 consecutive female patients with lymph node negative, ER+/HER2- breast cancers and low RS treated at our center during the study period (Table 1). The patient median age at breast cancer diagnosis was 57 years (range 22–90). Most patients (71.8%: 1002/1396) were >50 years old, 23.7% (331/1396) were between 40 and 49 years old, and 4.5% (63/1396) were <40 years old at the initial diagnosis of breast cancer. Of the 1396 tumors, 36.2% (505/1396) had a RS of 0 to 10, and 63.8% (891/1396) had a RS of 11–17. The median tumor size was 1.2 cm (range 0.3–5.8), and 21.3% (297/1396) were multifocal. Most tumors (77.1%; 1076/1396) were invasive ductal carcinoma (IDC) not otherwise specified, 13.5% (188/1396) were invasive lobular carcinoma (ILC), 5.2% (72/1396) were mixed ductal and lobular histology, and 4.3% (60/1396) were special histologic subtypes. LVI was identified in 19.1% (266/1396) of patients. All patients had a sentinel lymph node biopsy with negative nodes, including 74 patients with isolated tumor cells (ITCs), i.e. pN0(i+).

**Table 1:** Clinicopathologic characteristics of all 1396 patients with lymph node negative ER+/HER2- breast cancer and RS < 18 (all percentages within columns)

	RS 0–10 (*n* = 505)	RS 11–17 (*n* = 891)	Total (*n* = 1396)
RS: mean, median (range)	7, 8 (0–10)	14, 14 (11–17)	12, 12 (0–17)
Age at diagnosis			
Mean, median (range); years	58, 59 (25–84)	56, 55 (22–90)	57, 57 (22–90)
< 40 years; n (%)	21 (4.2%)	42 (4.7%)	63 (4.5%)
40–49 years; n (%)	111 (22%)	220 (24.7%)	331 (23.7%)
≥ 50 years; n (%)	373 (73.9%)	629 (70.6%)	1002 (71.8%)
Tumor size: mean, median (range); cm	1.31, 1.2 (0.4–5.8)	1.32, 1.2 (0.3–4.7)	1.32, 1.2 (0.3–5.8)
Multifocality; n (%)	123 (24.4%)	174 (19.5%)	297 (21.3%)
LVI; n (%)	90 (19.8%)	176 (17.8%)	266 (19.1%)
Local treatment; n (%)			
BCS only	15 (3%)	33 (3.7%)	48 (3.4%)
BCS and radiation therapy	319 (63.2%)	611 (68.6%)	930 (66.6%)
Total mastectomy	171 (33.9%)	243 (27.3%)	414 (29.7%)
Total mastectomy and radiation therapy	0	4 (0.4%)^b^	4 (0.3%)^b^
Systemic therapy; n (%)			
Endocrine therapy alone	462 (91.5%)	722 (81%)	1184 (84.8%)
Endocrine therapy and chemotherapy^a^	22 (4.4%)	147 (16.5%)^a^	169 (12.1%)^a^
No systemic therapy	21 (4.2%)	22 (2.5%)	43 (3.1%)
Median follow-up (range); months	52 (0.9–108.3)	52.2 (1–93)	52 (0.9–108.3)
LRR; n (%)	5 (1%)	8 (0.9%)	13 (0.9%)

*BCS* breast conserving surgery, *LRR* locoregional recurrence, *LVI* lymphovascular invasion, *RS* recurrence score.

^a^One patient did not complete endocrine therapy

^b^Radiation therapy following total mastectomy was given for ductal carcinoma in situ within 1 mm of the surgical margin (*n* = 3), and large (7 cm) tumor (*n* = 1)

Overall, 66.6% (930/1396) women were treated with breast conserving surgery (BCS) and radiation therapy, 3.4% (48/1396) with BCS alone, 29.7% (414/1396) with total mastectomy, and 0.3% (4/1396) with total mastectomy and radiation therapy. Most (96.9%; 1353/1396) patients received endocrine therapy, including 84.8% (1184/1396) treated with endocrine therapy alone and 12.1% (169/1396) treated with chemotherapy plus endocrine therapy. Only 3.1% (43/1396) received no systemic therapy.

Eight of 1396 (0.6%) patients developed distant metastases. None of these eight patients had LRR. One patient (0.1%) with distant metastases died of disease 64 months after her initial breast cancer diagnosis, six patients (0.4%) died of other causes, and six patients (0.4%) died of unknown causes.

### Patients with locoregional recurrence (LRR)

We recently demonstrated that RS is significantly associated with the risk of LRR in a large cohort of lymph node negative, ER+/HER2- breast cancer patients [21]. The risk of LRR was increased >4-fold (hazard ratio: 4.61, 95% CI 1.90–11.19, *p* < 0.01) and 3-fold (hazard ratio: 2.81, 95% CI 1.41–5.56, p < 0.01) for high and intermediate risk groups compared to the low risk group [21].

At a median follow-up of 52 months (range 0.9–108.3), 0.9% (13/1396) of patients with low RS developed LRR. The LRR occurred within five years of the index breast cancer diagnosis in 11 patients (Table 2). LRR was confirmed by pathologic examination of a biopsy specimen of the recurrent tumor tissue in all 13 patients. Sites of LRR included the ipsilateral breast (*n* = 8), chest wall (*n* = 3), axillary node (*n* = 1), and internal mammary node (*n* = 1). The index tumors of all 13 patients with LRR had been excised with negative margins (no ink on carcinoma). All patients were pN0, and none was pN0(i+). Of the 13 patients, four had a total mastectomy, seven had BCS with radiation therapy, and two (both >70 years old) had BCS alone. Eight patients were treated with endocrine therapy alone, four patients received combined endocrine therapy and chemotherapy, and one patient received no systemic therapy.

**Table 2:** Clinicopathologic characteristics of all 1396 patients of lymph node negative ER+/HER2- breast cancer with RS < 18 by LRR (all percentages within columns)

	No LRR (*n* = 1383)	LRR (*n* = 13)
RS		
Mean, median (range)	12, 12 (0–17)	11, 12 (0–17)
RS 0–10; n (%)	500 (36.2%)	5 (38.5%)
RS 11–17; n (%)	883 (63.8%)	8 (61.5%)
Age at diagnosis		
Mean, median (range); years	57, 57 (22–90)	55, 54 (35–79)
< 40 years; n (%)	61 (4.4%)	2 (15.4%)
40–49 years; n (%)	328 (23.7%)	3 (23.1%)
≥ 50 years; n (%)	994 (71.9%)	8 (61.5%)
Tumor size, median (range); cm	1.31, 1.2 (0.3–5.8)	1.5, 1.3 (0.5–3.5)
Multifocality; n (%)	291 (21%)	6 (46.2%)
LVI; n (%)	262 (18.9%)	4 (30.8%)
Local treatment; n (%)		
BCS only	46 (3.3%)	2 (15.4%)
BCS and radiation therapy	923 (66.7%)	7 (53.8%)
Total mastectomy	410 (29.6%)	4 (30.8%)
Total mastectomy and radiation therapy^b^	4 (0.3%)^b^	0
Systemic therapy; n (%)		
Endocrine therapy only	1176 (85%)	8 (61.5%)
Endocrine therapy and chemotherapy^a^	165 (11.9%)^a^	4 (30.8%)
No systemic therapy	42 (3%)	1 (7.7%)
Median follow-up (range); months	51.9 (0.9–108.3)	71.4 (43.2–86.8)
Time to LRR, median (range), months	–	36.5 (9.7–74.3)

*BCS* breast conserving surgery, *LRR* locoregional recurrence, *LVI* lymphovascular invasion, *RS* recurrence score

^a^One patient did not complete endocrine therapy

^b^Radiation therapy following total mastectomy was given for ductal carcinoma in situ within 1 mm of the surgical margin (*n* = 3), and large (7 cm) tumor (*n* = 1)

Of the 13 patients with LRR, five patients (38.5%) had a RS of 0 to 10, while eight patients (61.5%) were in the RS 11–17 group. None of the 13 patients with LRR was enrolled in the TAILORx or RxPONDER trials (see Discussion).

### Patients treated with adjuvant endocrine therapy only

Of the 1396 patients, most patients (84.8%; 1184/1396) were treated with adjuvant endocrine therapy and no chemotherapy (Table 3). Only 0.7% (8/1184) of patients developed LRR in this treatment group. All eight patients were alive at the last follow-up. Two of the eight patients with LRR were <40 years old at the initial diagnosis of breast cancer. All patients were pN0, and none was pN0(i+). Five patients had a family history of breast cancer, but none of the four patients who underwent genetic testing had germline BRCA1 or BRCA2 gene mutations (Table 4). All eight patients received standard local treatment. Three patients had LVI but no other significant risk factors for LRR were identified.

**Table 3:** Clinicopathologic characteristics of 1184 cases of lymph node negative ER+/HER2- breast cancer with RS < 18, treated with endocrine therapy only (all percentages within columns)

	RS 0–10 (*n* = 462)	RS 11–17 (*n* = 722)	Total (*n* = 1184)
RS: Mean, median (range)	7, 8 (0–10)	14, 14 (11–17)	11, 12 (0–17)
Age at diagnosis			
Mean, median (range); years	58, 59 (25–84)	58, 57 (22–90)	58, 58 (22–90)
< 40 years; n (%)	19 (4.1%)	21 (2.9%)	40 (3.4%)
40–49 years; n (%)	98 (21.2%)	156 (21.6%)	254 (21.5%)
≥ 50 years; n (%)	345 (74.7%)	545 (75.5%)	890 (75.2%)
Tumor size, median (range); cm	1.28, 1.1 (0.35–5.5)	1.28, 1.1 (0.3–4.5)	1.28, 1.1 (0.3–5.5)
Multifocality; n (%)	106 (22.9%)	128 (17.7%)	234 (19.8%)
LVI; n (%)	72 (15.6%)	126 (17.5%)	198 (16.7%)
Local treatment; n (%)			
BCS only	12 (2.6%)	28 (3.9%)	40 (3.4%)
BCS and radiation therapy	301 (65.2%)	515 (71.3%)	816 (68.9%)
Total mastectomy	149 (32.3%)	177 (24.5%)	326 (27.5%)
Total mastectomy and radiation therapy	0	2 (0.3%)	2 (0.2%)
LRR; n (%)	3 (0.6%)	5 (0.7%)	8 (0.7%)
Median follow-up (range); months	51.7 (0.9–108.3)	51.3 (1–93)	51.4 (0.9–108.3)

*BCS* breast conserving surgery, *LRR* locoregional recurrence, *LVI* lymphovascular invasion, *RS* recurrence score

**Table 4:** Clinicopathologic characteristics of eight patients with LRR in the patient cohort treated with endocrine therapy and no chemotherapy

Patients	#1	#2	#3	#4	#5	#6	#7	#8
Family history of breast cancer	No	Yes	Yes	Yes	Yes	Yes	No	No
BRCA mutations	Negative	Negative	Negative	Negative	Negative	Not tested	Not tested	Not tested
Tumor histotype	IDC, Grade 2	IDC, Grade 2	IDC, Grade 1	IDC, Grade 2	ILC, not graded	IDC, Grade 1	IDC, Grade 2	IDC, Grade 2
Tumor size (cm)	0.7	2.2	1.5	0.7	2.1	1.3	2	3.5
Multifocality	Yes	Yes	Yes	No	No	No	No	No
LVI	No	No	No	Yes	No	No	Yes	Yes
ER IHC (%)	100	80	90	98	98	100	95	98
PR IHC (%)	100	70	90	20	10	70	40	98
RS	0	14	7	16	14	4	17	12
ESR1 expression	11.2	10.4	9.8	9.8	10.2	12.1	11.5	11.8
PgR expression	>10	8.8	8.7	5.8	6.6	9.1	6.3	>10
Surgery	TM	TM	TM	BCS	BCS	BCS	BCS	BCS
Radiation therapy	No	No	No	Yes	Yes	No	Yes	No
Time to LRR (months)	42	65	29	50	37	41	24	46
Site of LRR	Chest wall	Internal mammary node	Chest wall	Ipsilateral breast	Ipsilateral breast	Ipsilateral breast	Ipsilateral breast	Ipsilateral breast

*BCS* breast conserving surgery, *ER* estrogen receptor, *ESR1* ER gene, *IDC* invasive ductal carcinoma, *IHC* immunohistochemistry, *ILC* invasive lobular carcinoma, *LRR* locoregional recurrence, *LVI* lymphovascular invasion, *PR* progesterone receptor, *PgR* PR gene, *RS* recurrence score, *TM* total mastectomy

## Discussion

As demonstrated by retrospective analysis of randomized clinical trials and non-randomized studies, the 21-gene RS assay is invaluable in guiding treatment recommendations for patients with early stage, ER+/HER2- breast cancer [8–17, 24]. Data from the NSABP (the National Surgical Adjuvant Breast and Bowel Project) B14 and B20 patients with node negative, ER+/HER2- breast cancer suggest that besides quantifying the likelihood of distant recurrence (prognostic value) within ten years of the initial diagnosis [3], the RS assay estimates the magnitude of chemotherapy benefit (predictive value) [4]. Recently, the RS risk categories have been reported to be associated with an increased risk of LRR [18–21]. Little is known about a subset of patients that develop LRR, especially in the low RS group. We recently documented a very low distant recurrence rate in node negative, ER+/HER2- breast cancer patients with low RS treated at our institution [25]. In this study, we report the rate of LRR and clinicopathologic characteristics of women with LRR in the same patient cohort.

Our study shows that >99% of women were free of LRR at a median follow up of 52 months. Only 0.9% (13/1396) developed LRR in this cohort of consecutive female patients. None of the 13 patients with LRR had distant metastases. Other studies reported similar low rates of LRR and breast cancer specific mortality in patients with low RS. A study of 163 patients showed that a RS > 24 was associated with LRR in patients treated with total mastectomy but not in those treated with BCS. Among women treated with total mastectomy, the five year LRR rate was 27.3% in patients with a RS > 24 versus 10.7% in patients with a RS ≤ 24 [19]. In another study, RS was a predictor of LRR along with age and treatment type in multivariate analysis [18]. Mamounas et al. reported a ten year LRR of 4.3% (95% CI, 2.3% to 6.3%) in tamoxifen treated patients with a low RS, and significant associations between RS and LRR in tamoxifen treated patients from NSABP B14 and B20 trials (*p* < 0.001), placebo treated patients from NSABP B14 trial (*P* = 0.022), and in chemotherapy plus tamoxifen treated patients from NSABP B20 trial (*P* = 0.028) [18].

An increased risk of LRR has been linked to a variety of clinicopathologic factors including patient age, tumor size and grade, LVI, the number of positive lymph nodes, bilateral breast cancer, ER/PR status, Ki67 proliferation index and the length of endocrine therapy [26–30]. Of the eight patients treated with endocrine therapy alone, three women had LVI on excision but no other significant risk factors for LRR were identified. Furthermore, all eight patients received standard local treatment.

The final results of two ongoing prospective studies, TailoRx (Trial Assigning IndividuaLized Options for treatment (Rx)) and RxPONDER (Rx for Positive Node, Endocrine Responsive breast cancer) [31–33] are not yet available. Notably, to minimize the risk of omitting chemotherapy, the TailoRx trial narrowed the low risk group to a RS of 0–10, expanded the intermediate risk group to include tumors with a RS of 11–25, and defined the high risk group as a RS ≥ 26 [31, 32]. Data from TailoRx for patients with RS 0–10 treated with hormonal therapy alone shows that 98.7% are free of distant recurrence or LRR at five years after the initial diagnosis of breast cancer [32]. In our cohort, the rate of LRR in the RS 0–10 group treated with only adjuvant endocrine therapy was 0.6% (3/462), consistent with the results of the TailoRx trial.

Our study cohort is unique as it consists of a large, consecutive population of women with low RS treated at a single institution with available clinical follow-up information. The 21-gene RS assay results were prospectively included and considered in the treatment planning for all patients. The main limitations of our study include its retrospective design and the low number of LRR events precluding a formal statistical analysis. In addition, our results may be less applicable to general patient populations as our tertiary academic institution predominantly treats women with screen detected breast cancer and women from a specific geographic region. The follow-up interval is less than five years in some patients due to the relatively recent implementation of the 21-gene RS assay. However, compared to our previous publication [25], we now report LRR rates at a longer median follow-up of 52 months.

## Conclusions

Our study of node negative, ER+/HER2- breast cancer patients with low RS (<18) observed extremely low rates of LRR: 0.9% (13/1396) in the whole cohort and 0.7% (8/1184) in patients treated with endocrine therapy alone. We report detailed clinicopathologic features of women who developed LRR in this low RS cohort.

## Abbreviations

BCS: Breast conserving surgery
ER: Estrogen receptor
IDC: Invasive ductal carcinoma
ILC: Invasive lobular carcinoma
ITC: Isolated tumor cells
LRR: Locoregional recurrence
LVI: Lymphovascular invasion
NSABP: The National Surgical Adjuvant Breast and Bowel Project
RS: Recurrence score
RT-PCR: Reverse transcriptase polymerase chain reaction
RxPONDER: Rx for Positive Node, Endocrine Responsive breast cancer
TailoRx: Trial Assigning IndividuaLized Options for treatment (Rx)
